# A Case of Hemorrhage before Delivery

**Published:** 1885-11

**Authors:** E. J. Doering

**Affiliations:** Late Surgeon U. S. M. H. Service, Chicago, Ill.


### A CASE OF HEMORRHAGE BEFORE DELIVERY.

*By E. J. Doering, M. D., late Surgeon U. S. M. H. Service, Chicago, Ill.*

For Daniel's Texas Medical Journal.

HEMORRHAGE before delivery from detachment of a normally situated placenta, so called accidental hemorrhage, is one of the dangerous complications of labor, though fortunately very rare.

In those cases where the blood flows between the membranes and ducidua and finds an exit externally, the diagnosis is easily established, and prompt measures can at once be instituted. But when the blood collects internally, the diagnosis becomes by no means easy, and the patient often goes into collapse before the true nature of the trouble is discovered, and dissolution prevents the attempt of surgical interference. Professor Goodell, of Philadelphia, has collected 106 cases of concealed hemorrhage before labor, out of which 54 died, nearly 50 per cent. Out of 107 children only seven were born alive. The mortality is far greater—both for mother and child—than it is in placenta prævia or in any other complication of labor. It is nevertheless true that the subject of concealed hemorrhage has not received that attention from authors and teachers which its importance deserves, and the writer believes that a more careful study of such cases would result in improved methods of treatment with much better results.

The symptom to which I attach most importance in an early recognition of concealed hemorrhage from premature detachment of the placenta, is the peculiar, agonizing and excruciating pain located in the womb, and which depends on the painful stretching of the latter by the retained coagula. Add to this symptom a weak, rapid, compressible pulse, a cool skin, absence of labor pains, and I think the diagnosis is clear enough to warrant immediate interference. To the latter I attribute the successful issue in the following case:

On the 4th of July of the present year Mrs. F. A. II-para, 31 years of age, in the ninth month of pregnancy, slipped in walking down stairs when within one step from the bottom of the stairs and fell on both knees. She sustained no injury of any kind and felt no particular pain at the time. During the night however she had pains in the right side of the womb simulating labor pains. These continuing, I was sent for and found on arrival the os dilated to the size of a nickel, the membranes very tense, and excruciating pain in the right side of the uterus. The pain being more or less continuous, and totally different from ordinary labor pain, the pulse being rather rapid and soft, skin cool, and the patient appearing pale and anxious, I suspected internal hemorrhage, particularly in view of the fall she had sustained. The membranes were therefore ruptured, followed by a gush of bloody fluid, the amniotic cavity being well filled therewith. The diagnosis being now clear, the uterus was held firmly compressed, and stimulated to contraction. The os was rapidly dilated manually, and within one hour the patient was delivered of an apparently stillborn child, which, however, after twenty minutes of hard work was made to breathe and is doing well. The expulsion of the child was immediately followed by the discharge of the placenta together with a large mass of blood clots. Firm compression of the uterus, administration of ergot, and a binder prevented further hemorrhage. Examination of the placenta, showed a marginal insertion of the cord, and fully one-third of the placenta had evidently become prematurely detached as proven by the firm adherence of a solid clot over that extent of surface. With the best of nursing, absolute rest and carefully regulated diet, the patient rallied, and to-day is in the best of health.

